# Application of two-dimensional speckle-tracking echocardiography in radiotherapy-related cardiac systolic dysfunction and analysis of its risk factors: a prospective cohort study

**DOI:** 10.1186/s12872-024-03981-1

**Published:** 2024-06-27

**Authors:** Rong Liu, Li ang Xu, Ziqi Zhao, Ruoling Han

**Affiliations:** 1 Ultrasound department of the fourth hospital of Hebei Medical University, No.12 of Jiankang Road, Shijiazhuang, Hebei Provence China; 2 Radiotherapy department of the fourth hospital of Hebei Medical University, No.12 of Jiankang Road, Shijiazhuang, Hebei Provence China

**Keywords:** Two-dimensional speckle-tracking echocardiography, Radiotherapy, Cardiac systolic dysfunction, Mean heart dose

## Abstract

**Background:**

The cardiac toxicity of radiotherapy (RT) can affect cancer survival rates over the long term. This has been confirmed in patients with breast cancer and lymphoma. However, there are few studies utilizing the two-dimensional speckle-tracking echocardiography (2D-STE) to evaluate the risk factors affecting radiation induced heart disease (RIHD), and there is a lack of quantitative data. Therefore, we intend to explore the risk factors for RIHD and quantify them using 2D-STE technology.

**Methods:**

We ultimately enrolled 40 patients who received RT for thoracic tumors. For each patient, 2D-STE was completed before, during, and after RT and in the follow up. We analyzed the sensitivity of 2D-STE in predicting RIHD and the relationship between RT parameters and cardiac systolic function decline.

**Results:**

Left ventricle global longitudinal strain (LVGLS), LVGLS of the endocardium (LVGLS-Endo), LVGLS of the epicardium (LVGLS-Epi), and right ventricle free-wall longitudinal strain (RVFWLS) decreased mid- and post-treatment compared with pre-treatment, whereas traditional parameters such as left ventricular ejection fraction (LVEF), cardiac Tei index (Tei), and peak systolic velocity of the free wall of the tricuspid annulus (s’) did not show any changes. The decreases in the LVGLS and LVGLS-Endo values between post- and pre-treatment and the ratios of the decreases to the baseline values were linearly correlated with mean heart dose (MHD) (all *P* values < 0.05). The decreases in the LVGLS-Epi values between post- and pre-treatment and the ratios of the decreases to the baseline values were linearly correlated with the percentage of heart volume exposed to 5 Gy or more (V5) (*P* values < 0.05). The decrease in RVFWLS and the ratio of the decrease to the baseline value were linearly related to MHD and patient age (all *P* values < 0.05). Endpoint events occurred more frequently in the right side of the heart than in the left side. Patients over 56.5 years of age had a greater probability of developing right-heart endpoint events. The same was true for patients with MHD over 20.2 Gy in both the left and right sides of the heart.

**Conclusions:**

2D-STE could detect damages to the heart earlier and more sensitively than conventional echocardiography. MHD is an important prognostic parameter for LV systolic function, and V5 may also be an important prognostic parameter. MHD and age are important prognostic parameters for right ventricle systolic function.

## Background

Currently, the left ventricular ejection fraction (LVEF) is the most stable and reliable parameter for evaluating cardiac function in radiation induced heart disease (RIHD). However, LVEF remains within the normal range due to compensation in the subclinical stage, so it cannot detect early myocardial dysfunction. Therefore, it may not be the best parameter to detect subclinical myocardial injury [1].

Speckle tracking echocardiography (STE) offers measurement parameters that enhance the ability to detect and quantify subtle changes in myocardial deformation patterns [2, 3]. Existing 2D-STE techniques have the potential to detect early RIHD, but there are few studies utilizing 2D-STE to evaluate the risk factors, and there is a lack of quantitative data [4]. Therefore, we aimed to explore the risk factors for RIHD and quantify them using 2D-STE technology.

## Methods

### Research participants

We enrolled 58 patients aged ≥18 years who received RT for thoracic tumors from May 2021 to May 2022. The median age is 63 years, with a range of 37-81 years. All patients signed the informed-consent form for our study. 2D-STE was completed before the start of RT, during RT (when half the prescribed dose had been administered), and within 1–2 days of the end of RT. Exclusion criteria were patients: (I) who had received radiation to the heart as part of RT before this treatment, (II) who had received anthracycline chemotherapy drugs or targeted drug therapy before or during the study period, (III) with heart failure, (IV) with continuous atrial fibrillation, (V) with interventricular-conduction disorder, (VI) with significant valvular heart diseases, (VII) whose quality of 2D-STE images did not meet our standards for image diagnosis and analysis, (VIII) who could not complete all prescribed doses of RT, and (IX) who could not complete all 2D-STE examinations.

This study was approved and filed by the ethics committee of our institution.

### Instruments

In this study we used a GE Vivid E95 Color Doppler Ultrasound System (GE Healthcare, Chicago, IL, USA) equipped with an M5S probe (frequency, 1.7–3.3 MHz), an Echo Picture Archiving and Communications (PAC) workstation with GE Echo 204 (GE Healthcare, Chicago, IL, USA).

### Collection of general information

Before performing 2D-STE on the patients, we recorded their general information, which is listed in Table 1.

**Table 1:** Basic information of patients

Item	Number of patients (proportion)
Gender	
Male	22 (55.00%)
Female	18 (45.00%)
Age (years)	
≤56.5	16 (40.00%)
>56.5	24 (60.00%)
Disease composition	
Lung cancer	18 (45.00%)
Esophageal cancer	11 (27.50%)
Breast cancer	10 (25.00%)
Thymic carcinoma	1 (2.50%)
Decreased heart function	
Left heart	7 (17.50%)
Right heart	10 (25.00%)

### Image acquisition

#### Conventional echocardiography

The patient lay in the left-lateral decubitus position, breathing calmly, and was connected to the ECG synchronously.

Two-dimensional ultrasound parameters were measured as follows: (1) LVEF, using the apical-biplane Simpson method; (2) mitral annular-plane systolic excursion (MAPSE); (3) tricuspid annular plane systolic excursion (TAPSE), using M-mode at the annulus of the lateral tricuspid valve; and (4) right ventricular (RV) fractional-area change (FAC) in an apical four-chamber view dominated by the right ventricle (RV).

#### Pulse and tissue Doppler ultrasound parameters

Pulse and tissue Doppler ultrasound parameters were as follows: (1) Doppler image of the tissue of mitral valve annulus at the left ventricular (LV) lateral wall and the ventricular septum, (2) Doppler image of the tissue of tricuspid valve annulus at the free wall, (3) (calculated) left- and right-heart Tei index, and (4) peak systolic velocity (PSV) of the free wall of the tricuspid annulus (s’).

#### Two-dimensional speckle-tracking echocardiography

**Fig. 1:**
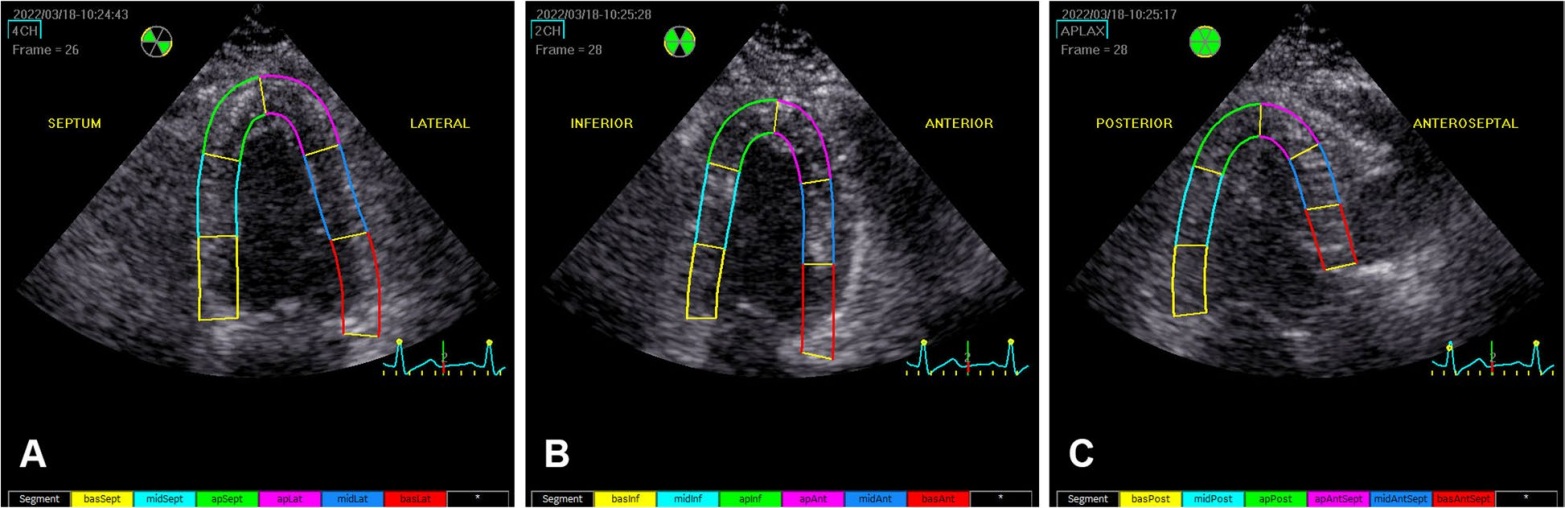
Echo PAC endocardium automapping interface: **A** Four-chamber view. **B** Two-chamber view. **C** Three-chamber view

**Fig. 2:**
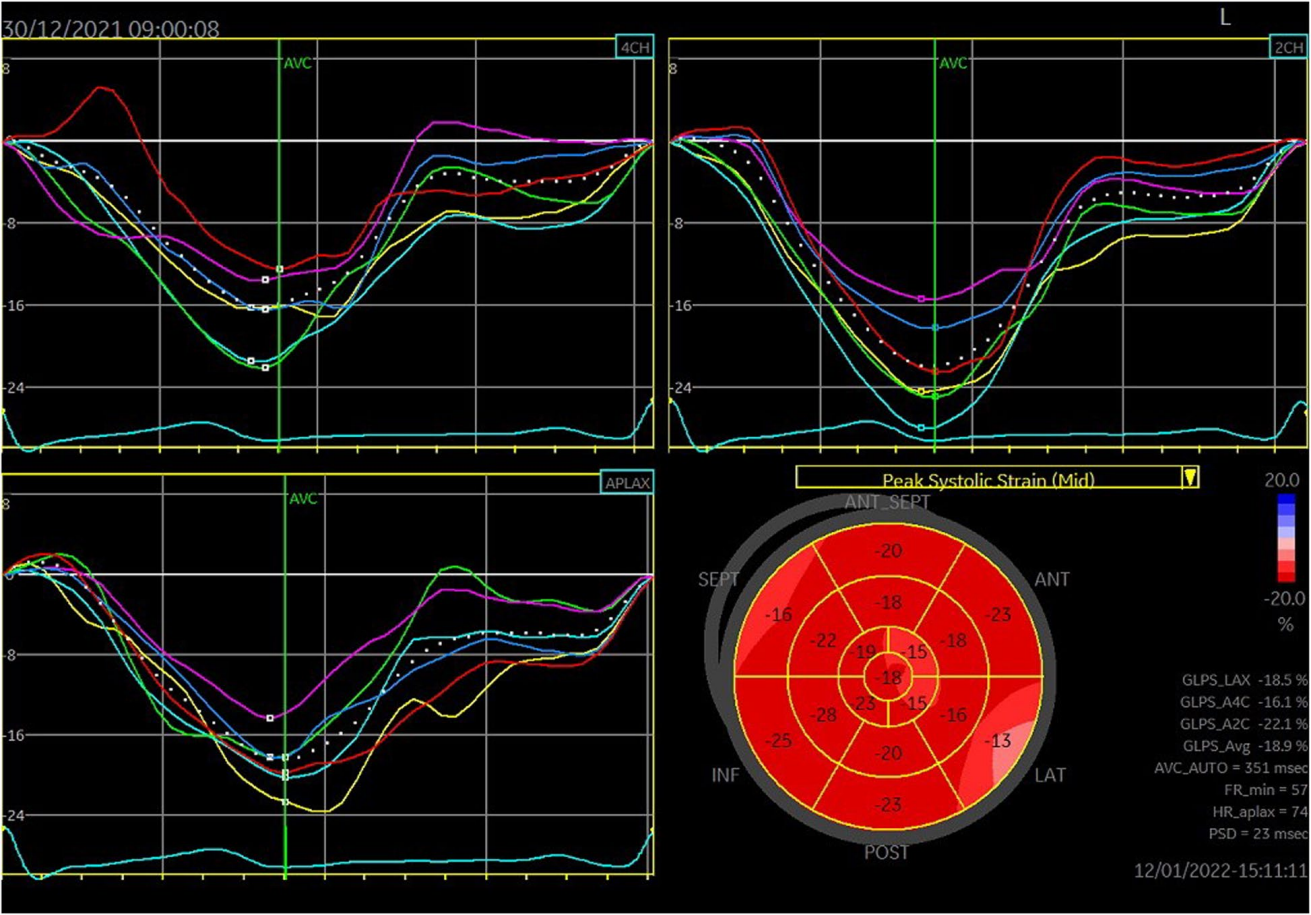
Schematic diagram of left ventricular time-strain curve

**Fig. 3:**
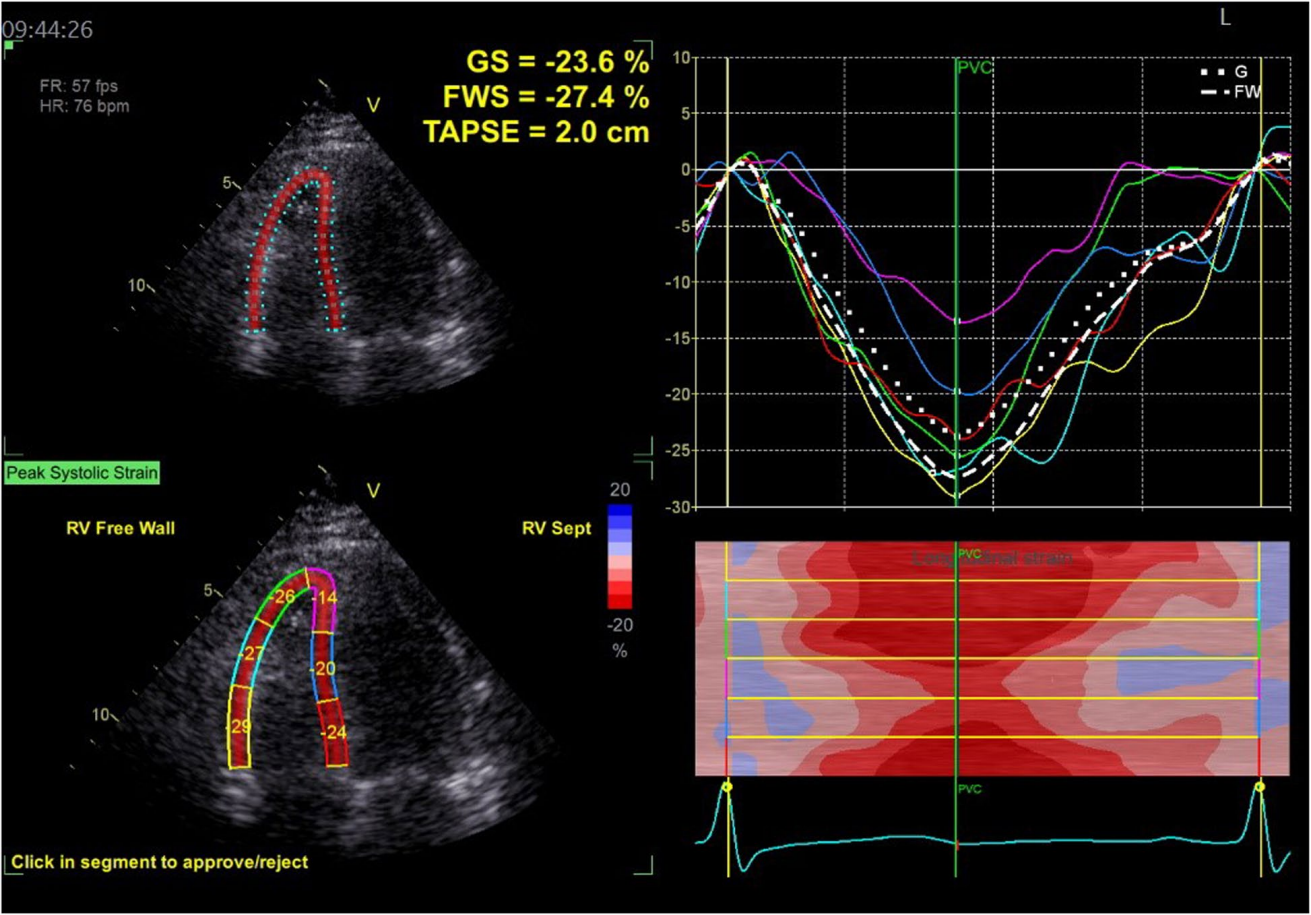
Schematic diagram of right ventricular time-strain curve

Dynamic images were stored in standard apical four-, two-, and three-chamber views, as well as in the right-sided four-chamber view. We recorded dynamic images of at least three cardiac cycles per section (view). We adjusted depth and width so that the image included the entire LV or full extent of the RV. After dynamic images were transmitted to the Echo PAC workstation, the endocardium was traced using GE Echo 204, which automatically analyzed the time–strain curve of each segment on each section, and obtained LVGLS-mid, -endo and -epi (Figs. 1, 2 and 3).

### Determination of endpoint events

During follow-up, a patient was considered to demonstrate cardiac damage if one of the following occurred: (a) a ≥5% decrease in LVEF from baseline to an absolute value of <53%, with symptoms or signs of heart failure; or (b) a ≥10% decrease in LVEF from baseline to an absolute value of <53% without symptoms or signs of heart failure [5], (c) RVFAC <35%, or (d) PSV of the tricuspid valve at the RV free wall (s’) <10 cm/s [6]; (2) cardiac death; (3) myocardial infarction; or (4) unstable angina.

### Statistical analysis

We determined the distribution characteristics of the data (normal or skewed) using the Kolmogorov–Smirnov test. Normally distributed data are presented as mean ± standard deviation (SD). Based on these distribution characteristics, we used repeated-measures analysis of variance (ANOVA) to compare differences in 2D-STE and conventional echocardiography parameters at baseline, mid-radiation, and post-radiation. Correlates of cardiotoxicity were assessed using multiple linear regression and correlation analyses. We analyzed significant variables for power using receiver operating curves (ROCs) and determined the cutoff values using the Youden index (YI). The Kaplan–Meier method was used to analyze and follow up on the -endpoint events of patients, and the log-rank method was used to compare endpoint events differences between patients in different groups. We performed all analyses using SPSS version 21.0 (IBM Corp., Armonk, NY, USA). Two-sided *P* < 0.05 was considered statistically significant.

## Results

### General information

Of the 58 total patients, 3 could not complete RT, 9 had 2D-STE image quality that did not meet the study requirements, and 6 could not complete all 2D-STE examinations. We ultimately included the remaining 40 patients; 22 were male, and median age was 61 years (range, 35–79 years). Detailed information on the patients’ disease composition is shown in Table 1. Median follow-up time for all patients was 82 days (range, 31–301 days). Seventeen patients (42.50%) developed left- or right-heart function decline.

**Fig. 4:**
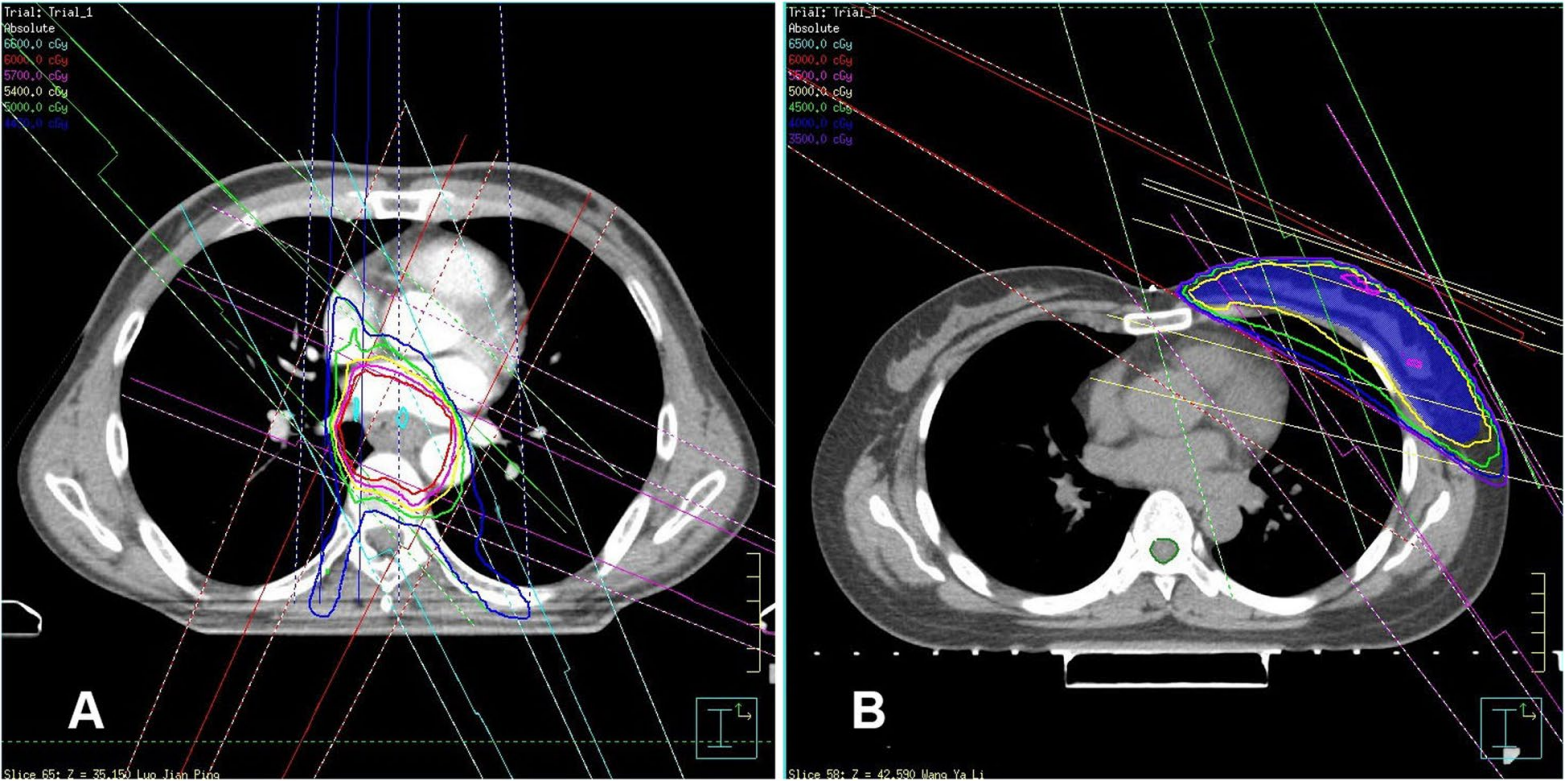
Registry volume curve and field distribution of radiotherapy plans for different patients. A: Esophageal cancer patient. B: Breast cancer patient

**Fig. 5:**
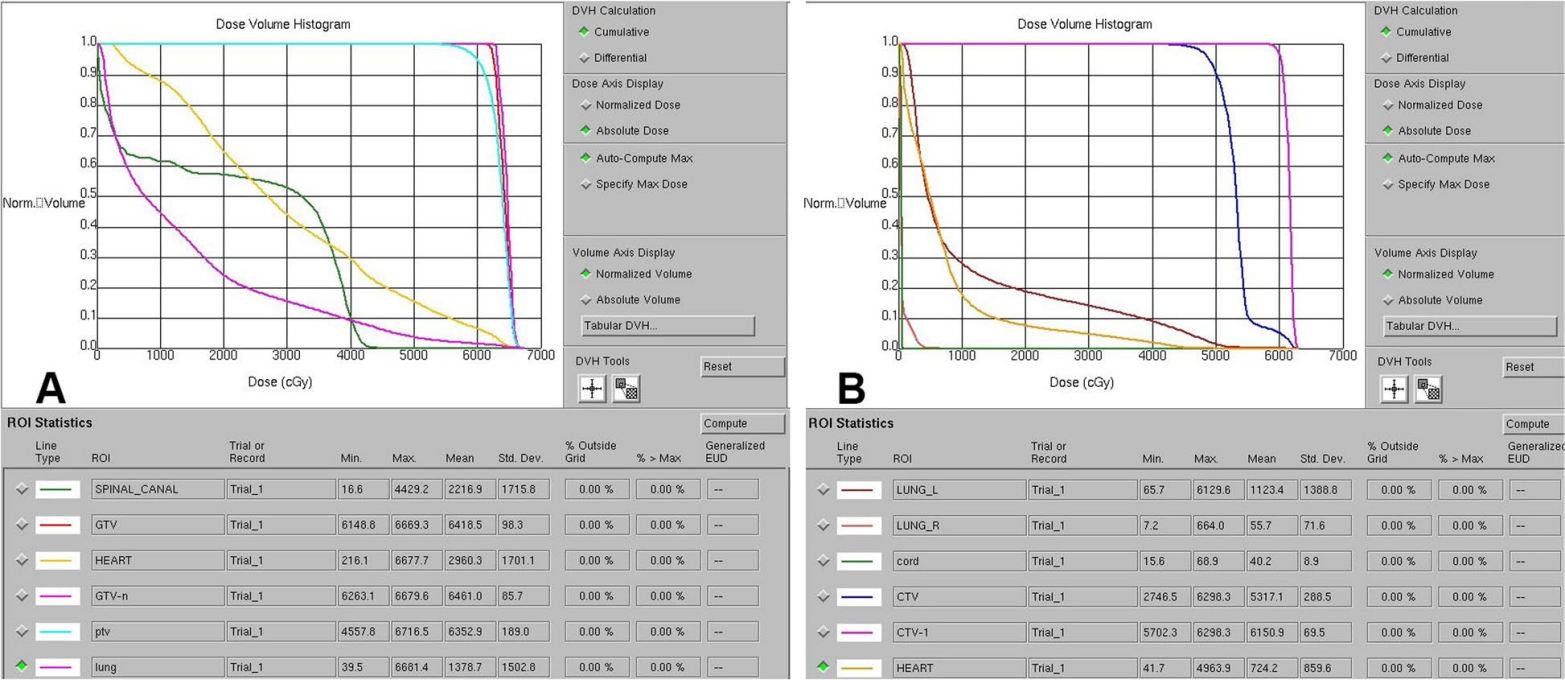
Radiation dose–volume histograms for different patients: A: Esophageal cancer patient. B: Breast cancer patient

We calculated each patient’s radiation dose parameters according to the dose–volume histogram (DVH) of that patient’s RT plan (Figs. 4 and 5). The results are shown in Table 2.

**Table 2:** Results of various parameters of radiotherapy

Radiation therapy parameter	Result
Heart volume (cm^3^)	695.99 ± 194.87
Prescribed dose (Gy)	57.83±44.0.9
Maximum heart dose (Gy)	48.26±22.31
Mean heart dose (Gy)	13.89±10.35
V5 (%)	49.62±33.95
V10 (%)	39.05±32.84
V20 (%)	25.65±23.2
V30 (%)	16.05±14.61
V40 (%)	9.69±10.09
V50 (%)	4.66±5.18

*Vx* Heart volume exposed to x Gy or more / Total heart volume × 100%.

### Echocardiographic parameters

#### Comparison of left ventricular systolic-function parameters

The LV systolic-function parameter LVEF had no statistical significance in the pre-treatment, mid-treatment, and post-treatment stages. The LV Tei index increased slightly in all three stages. The 2D strain parameters LV global longitudinal strain (LVGLS), LVGLS of the endocardium (LVGLS-Endo), and LVGLS of the epicardium (LVGLS-Epi) decreased mid- and post-treatment compared with pre-treatment (Table 3).

**Table 3:** Echocardiographic parameters

Echocardiographic parameter	Baseline	During treatment	After treatment	*P*
Left heart				
Left heart Tei index	0.35 ± 0.03	0.37 ± 0.04	0.37 ± 0.04	0.003
LVEF (%)	62.20 ± 6.00	61.75 ± 5.89	60.43 ± 5.91	0.081
LVGLS (%)	−18.85 ± 3.00	−16.83 ± 2.99	−16.35 ± 3.36	0.000
LVGLS-Endo (%)	−21.52 ± 3.44	−19.25 ± 3.42	−18.50 ± 3.76	0.000
LVGLS-Eepi (%)	−16.78 ± 2.83	−14.43 ± 2.92	−13.21 ± 6.11	0.000
Right heart				
Right heart Tei index	0.38 ± 0.06	0.39 ± 0.06	0.39 ± 0.06	0.280
RVFAC (%)	51.75 ± 6.55	45.48 ± 4.67	42.25 ± 9.03	0.000
RVFWLS (%)	−28.86 ± 1.26	−25.69 ± 2.29	−22.01 ± 5.07	0.000
s’ (cm/s)	13.23 ± 2.78	12.74 ± 2.96	13.04 ± 3.13	0.563
TAPSE (mm)	2.27 ± 0.42	2.07 ± 0.32	2.03 ± 0.45	0.013

*LVEF* Left ventricular ejection fraction, *LVGLS* Left ventricular global longitudinal strain, *LVGLS-Endo* Left ventricular global longitudinal strain of endocardium, *LVGLS-Epi* Left ventricular global longitudinal strain of epicardium, *RVFAC* Right ventricular fractional-area change, *RVFWLS* Right ventricular free-wall longitudinal strain, *TAPSE* Tricuspid annular plane systolic excursion, *s’* Peak systolic velocity of the free wall of the tricuspid annulus, *TAPSE* Tricuspid annular plane systolic excursion

#### Comparison of right ventricular systolic-function parameters

Both TAPSE and RVFAC decreased mid- and post-treatment compared with pre-treatment. The 2D strain parameter right ventricular free-wall longitudinal strain (RVFWLS) decreased mid- and post-treatment compared with pre-treatment (Table 2). Traditional detection values such as right-heart Tei index and S’ did not show any changes (Table 3).

#### Decreased value and percentage of left ventricular two-dimensional systolic-function parameters as shown by multiple linear analyses

We included the decreased values of LV 2D systolic-function parameters post- and pre-treatment, their percentages, and RT parameters that could be associated factors (patient age, heart volume, prescribed dose, maximum heart dose, mean heart dose (MHD), percentage of heart volume exposed to 5 Gy or more (V5), V10, V20, V30, V40, and V50) in multiple linear regressions. We found that ΔLVGLS_post-treatment–pretreatment_ (the difference between post- and pre-treatment), ΔLVGLS_post-treatment–pretreatment_/LVGLS_pretreatment_, ΔLVGLS-Endo_post-treatment–pretreatment_, and ΔLVGLS-Endo_post-treatment–pretreatment_/LVGLS-Endo_pretreatment_ were linearly correlated with MHD (Tables 4, 5, 6, and 7); and ΔLVGLS-Epi_post-treatment–pretreatment_ and ΔLVGLS-Epi_post-treatment–pretreatment_/LVGLS-Epi_pretreatment_ were linearly correlated with V5 (Tables 8 and 9).

**Table 4:** Multiple linear regressions of ΔLVGLS_post-treatment–pretreatment_ and its associated factors

Model	Unstandardized coefficient		Standardized coefficient	t	*P*	95.0% confidence interval for B	
	B	Standard error	Beta			Lower limit	Upper limit
(Constant)	−.023	.844		−.028	.978	−1.733	1.686
MHD	.002	.000	.514	3.649	.001	.001	.003

*LVGLS* Left ventricular global longitudinal strain, *MHD* Mean heart dose

**Table 5:** Multiple linear regressions of ΔLVGLS_post-treatment–pretreatment_/LVGLS_pretreatment_ and its associated factors

Model	Unstandardized coefficient		Standardized coefficient	t	*P*	95.0% confidence interval for B	
	B	Standard error	Beta			Lower limit	Upper limit
(Constant)	−44.317	19.755		−2.243	.031	−84.383	−4.251
MHD	.007	.001	.546	4.469	.000	.004	.010

*LVGLS* Left ventricular global longitudinal strain, *MHD* Mean heart dose

**Table 6:** Multiple linear regressions of ΔLVGLS-Endo_post-treatment–pretreatment_ and its associated factors

Model	Unstandardized coefficient		Standardized coefficient	t	*P*	95.0% confidence interval for B	
	B	Standard error	Beta			Lower limit	Upper limit
(Constant)	−.230	.911		−.253	.802	−2.077	1.616
MHD	.002	.001	.582	4.355	.000	.001	.003

*LVGLS-Endo* Left ventricular global longitudinal strain of endocardium, *MHD* Mean heart dose

**Table 7:** Multiple linear regressions of ΔLVGLS-Endo_post-treatment–pretreatment_/LVGLS-Endo_pretreatment_ and its associated factors

Model	Unstandardized coefficient		Standardized coefficient	t	*P*	95.0% confidence interval for B	
	B	Standard error	Beta			Lower limit	Upper limit
(Constant)	−45.911	18.431		−2.491	.017	−83.290	−8.532
MHD	.008	.001	.626	5.704	.000	.005	.011

*LVGLS-Endo* Left ventricular global longitudinal strain of endocardium

**Table 8:** Multiple linear regressions of ΔLVGLS-Epi_post-treatment–pretreatment_ and its associated factors

Model	Unstandardized coefficient		Standardized coefficient	t	*P*	95.0% confidence interval for B	
	B	Standard error	Beta			Lower limit	Upper limit
(Constant)	−40.481	18.962		−2.135	.042	−79.387	−1.574
V5	.535	.182	2.930	2.940	.007	.162	.908

*LVGLS-Epi* Left ventricular global longitudinal strain of epicardium, *V5* Percentage of heart volume exposed to 5 Gy or more

**Table 9:** Multiple linear regressions of ΔLVGLS-Epi_post-treatment–pretreatment_/LVGLS-Epi_pretreatment_ and its associated factors

Model	Unstandardized coefficient		Standardized coefficient	t	*P*	95.0% confidence interval for B	
	B	Standard error	Beta			Lower limit	Upper limit
(Constant)	−237.571	91.724		−2.590	.015	−425.774	−49.369
V5	2.970	.879	3.041	3.377	.002	1.166	4.775

*LVGLS-Epi* Left ventricular global longitudinal strain of epicardium, *V5* Percentage of heart volume exposed to 5 Gy or more

#### Decreased value and percentage of major right ventricular systolic-function parameters as shown by multiple linear analyses

We included the decreased values of RV systolic-function parameters post- and pre-treatment, their percentages, and the RT parameters that might have been associated factors (patient age, heart volume, prescribed dose, maximum heart dose, MHD, V5, V10, V20, V30, V40, and V50) in multiple linear regressions. We found that ΔRVFWLS _post-treatment–pretreatment_ was linearly related to MHD and patient age (Table 10), while ΔRVFWLS _post-treatment–pretreatment_/ RVFWLS _pretreatment_ was linearly related to MHD and patient age (Table 11).

**Table 10:** Multiple linear regressions of ΔRVFWLS _post-treatment–pretreatment_ and its associated factors

Model	Unstandardized coefficient		Standardized coefficient	t	*P*	95.0% confidence interval for B	
	B	Standard error	Beta			Lower limit	Upper limit
(Constant)	−5.568	3.177		−1.753	.088	−12.011	.875
MHD	.004	.001	.737	7.408	.000	.003	.005
Age	.115	.053	.215	2.157	.038	.007	.224

*RVFWLS* Right ventricular free-wall longitudinal strain, *MHD* Mean heart dose

**Table 11:** Multiple linear regressions of ΔRVFWLS _post-treatment–pretreatment_/ RVFWLS _pretreatment_ and its associated factors

Model	Unstandardized coefficient		Standardized coefficient	t	*P*	95.0% confidence interval for B	
	B	Standard error	Beta			Lower limit	Upper limit
(Constant)	−15.231	9.626		−1.582	.122	−34.755	4.292
MHD	.013	.002	.750	7.807	.000	.009	.016
Age	.368	.162	.218	2.271	.029	.039	.696

*RVFWLS* Right ventricular free-wall longitudinal strain, *MHD* Mean heart dose

### Cutoffs for age and mean cardiac dose

According to the YI, cutoff values for age and MHD were 56.5 years and 20.20 Gy, respectively.

### Follow-up results

The types of endpoint events, as the Determination of endpoint events section listed, that occur in all enrolled patients throughout the entire treatment and follow-up period include: (1) (b) a ≥10% decrease in LVEF from baseline to an absolute value of <53% without symptoms or signs of heart failure, (1) (c) RVFAC <35% and (1) (d) PSV of the tricuspid valve at the RV free wall (s’) <10 cm/s. No other endpoint event types appeared.

**Fig. 6:**
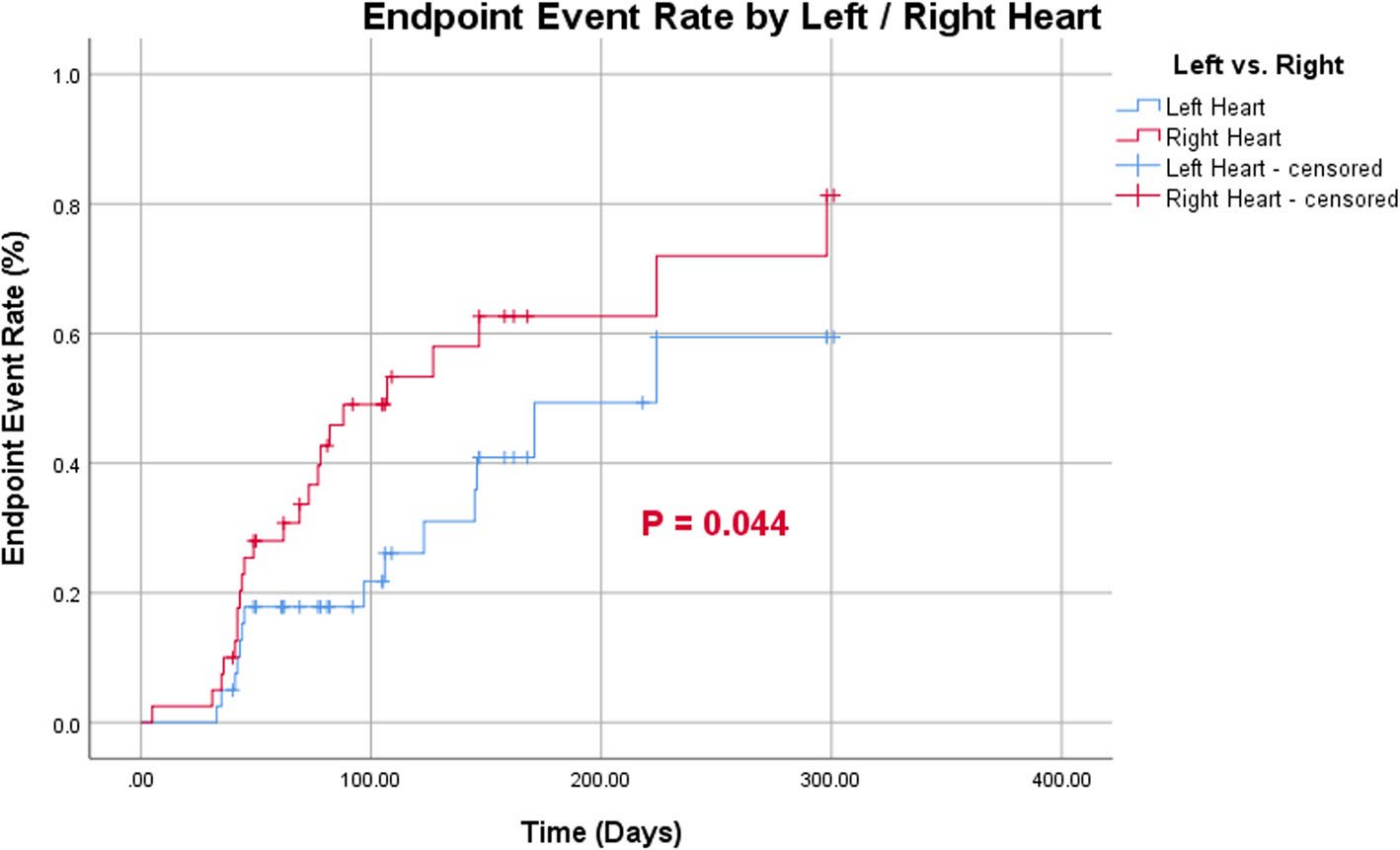
Endpoint event rate of left- and right-heart

**Fig. 7:**
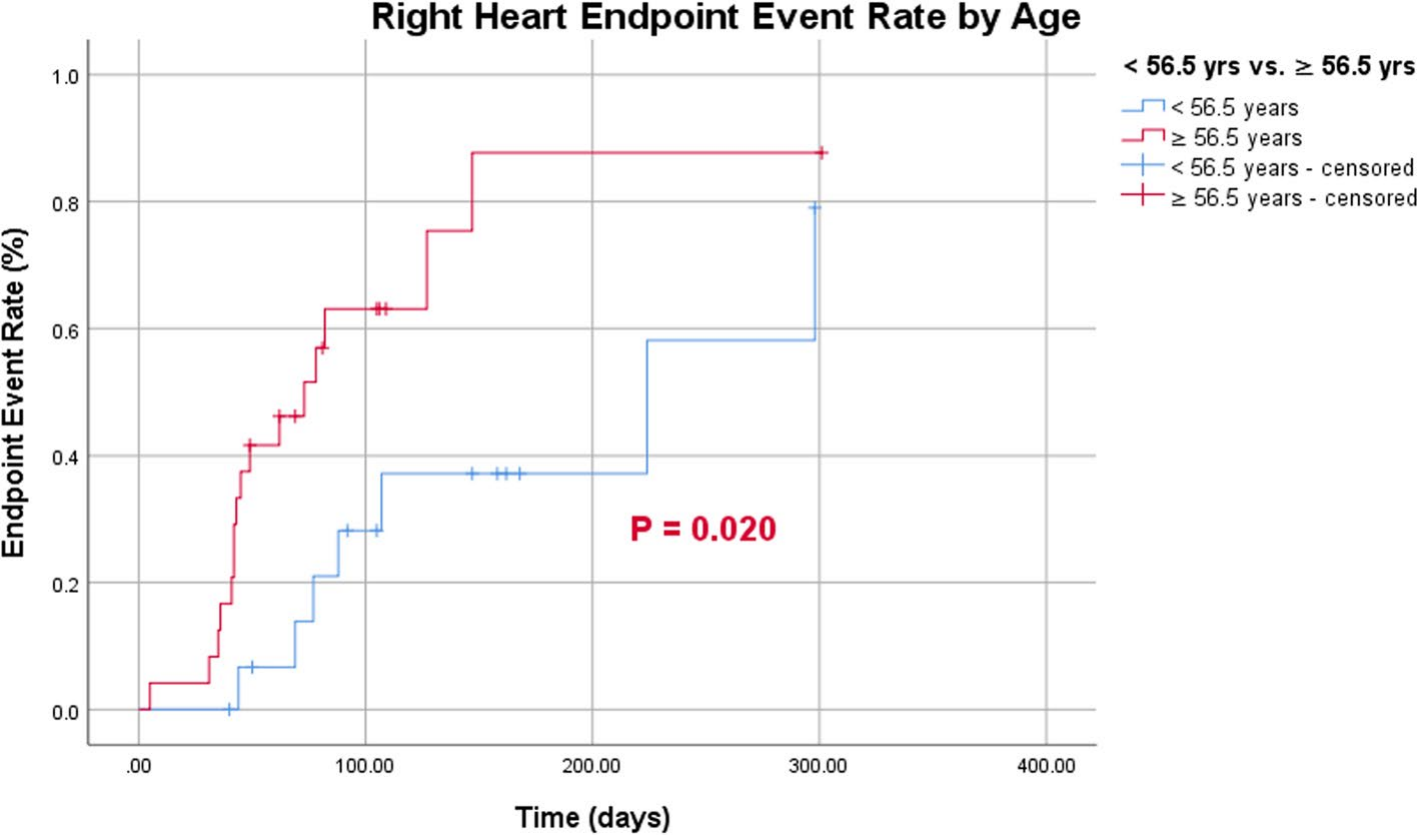
Endpoint event rate of right-heart by age

**Fig. 8:**
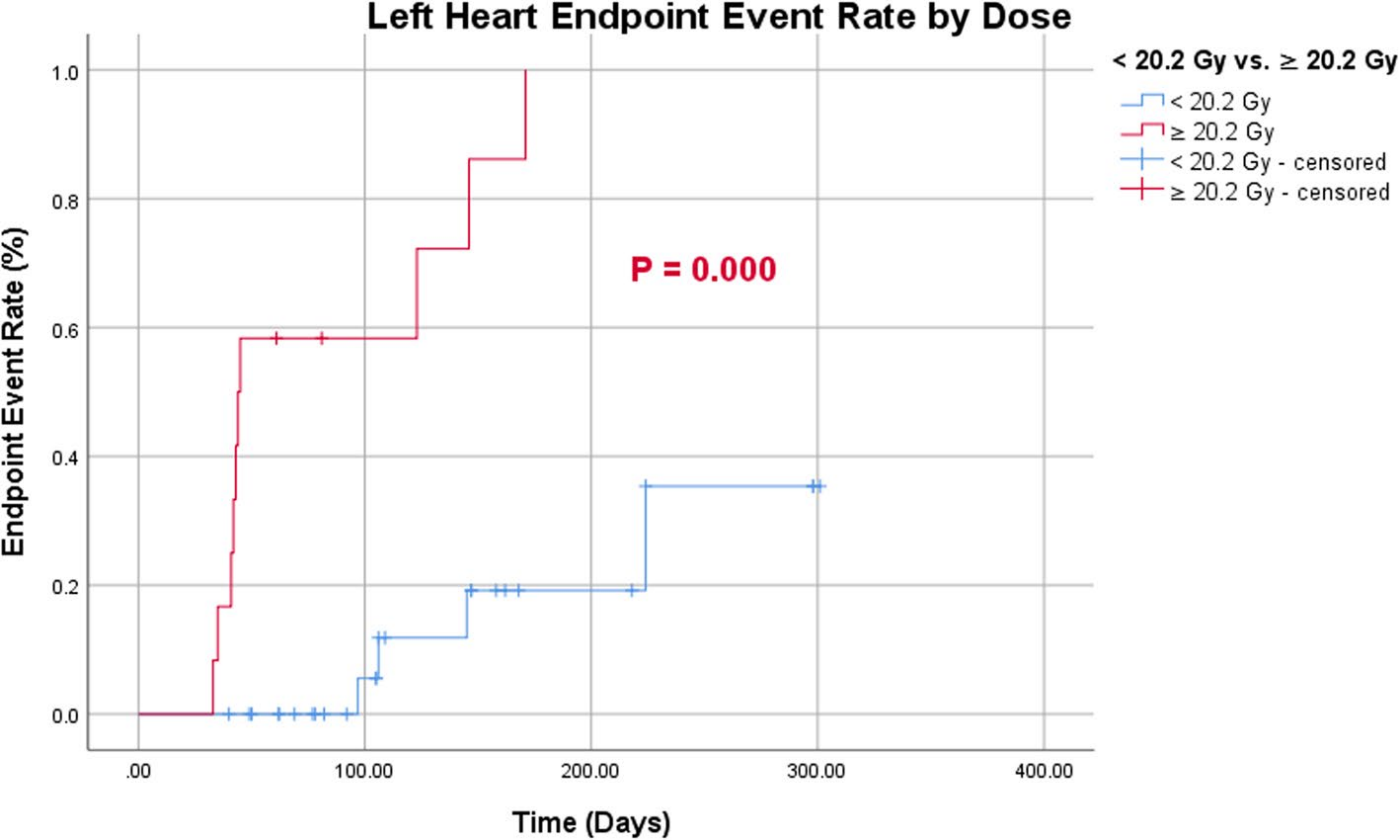
Endpoint event rate of left-heart by dose

**Fig. 9:**
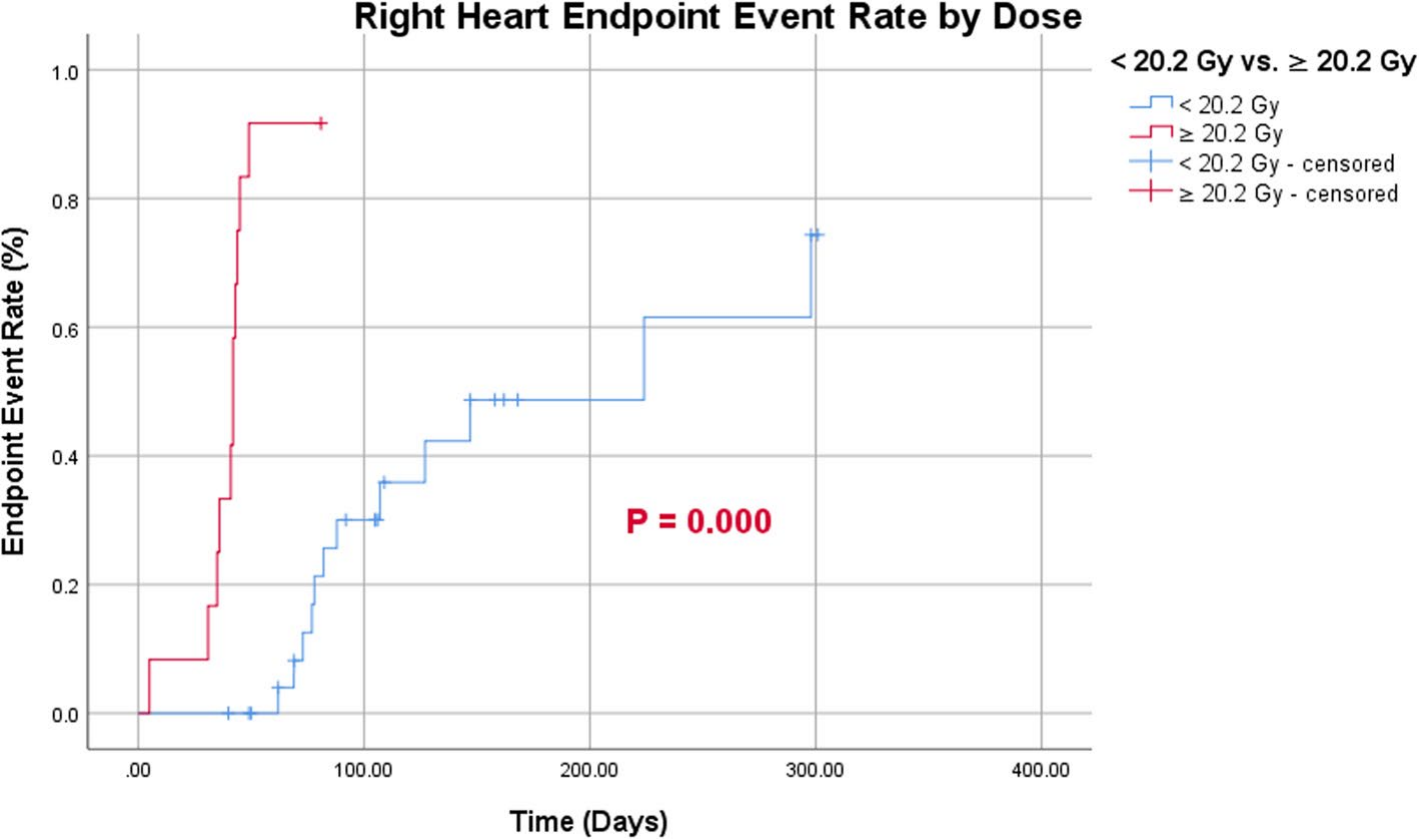
Endpoint event rate of right-heart by dose

Endpoint events occurred more frequently in the right heart than in the left (Fig. 6). According to the cutoff values for age and MHD, patients > 56.5 years of age had a greater probability of developing right-heart endpoint events than those < 56.5 years of age (Fig. 7). Patients with MHD > 20.20 Gy had a greater probability of endpoint events in both the left and right heart than those with a MHD of < 20.20 Gy (Figs. 8 and 9).

## Discussion

Thoracic tumors (including breast tumors) often require RT, during which the heart inevitably receives a certain dose of radiation. In the 1990s, during long-term follow-up of patients who had received mediastinal RT for Hodgkin lymphoma, Lee found that the relative risk (RR) of cardiac complications was 2.2–7.2 [7]. In patients who had received RT for esophageal cancer, the incidence of symptomatic cardiac injury was 10.8%, that of asymptomatic cardiac injury was 5–80%, and the interval between related events after RT was shorter than that in lymphoma patients [8].

Assessment of LV function is very important, however, LVEF also has its shortcomings: (I) it provides only indirect evaluation and cannot directly measure myocardial contractile function; (II) it is easily affected by many additional factors such as load conditions and heart rate; and, most importantly, (III) it is not sensitive enough to detect subtle changes in systolic function. LVGLS has proven to be a more sensitive indicator of systolic function than LVEF [2, 3].

In a study by Mark et al. [9], resting-gated single-photon emission computed tomography (SPECT) cardiac perfusion scans were used to provide objective, quantitative data on LV regional myocardial perfusion, regional wall motion, and EF. Abnormal myocardial perfusion occurred in 27%, 29%, 38%, and 42% of patients at 6, 12, 18, and 24 months after RT, respectively. This confirms that development of myocardial-perfusion abnormalities is related to interval of time after RT. In Hancock et al.’s study [10], their patients had evidence of ischemia by cardiovascular magnetic-resonance imaging but were clinically asymptomatic. Those authors speculated that the follow-up time might have been too short, and that over the course of a longer follow-up, symptoms may develop. No symptomatic coronary events occurred in any of our patients during follow-up, which may be due to the short follow-up time too. We know that complex left ventricular myocardial deformation is caused by the complex muscle fibers arrangement in the left ventricular wall. The myocardium is composed of three layers of muscle fibers arranged and moving in different directions, which coordinate and complement each other. Echopac software can automatically detect and identify layered myocardium, and obtain corresponding strain parameters. In our study, the 2D strain parameters LVGLS, LVGLS-Endo, LVGLS-Epi, and RVFWLS were more sensitive than the traditional parameters LVEF, RV cardiac Tei index, and peak systolic velocity of the free wall of the tricuspid annulus (s’) in detecting decreases in systolic function in the middle and end stages of RT. Therefore, 2D-STE can sensitively detect the changes in myocardial systolic function in subclinical stages.dd

We included the decreased values of the 2D systolic-function parameters post- and pre-treatment, the decrease in value as a percentage of pre-treatment value, and the RT parameters that might have been associated factors (patient age, heart volume, prescribed dose, maximum heart dose, MHD, baseline LVEF, V5, V10, V20, V30, V40, and V50) in multiple linear regressions. We found that ΔLVGLS_post-treatment–pretreatment,_ ΔLVGLS_post-treatment–pretreatment_/LVGLS_pre-treatment_, ΔLVGLS-Endo_post-treatment–pretreatment_, and ΔLVGLS-Endo_post-treatment–pretreatment_/LVGLS-Endo_pre-treatment_ were linearly correlated with MHD. Darby et al. proposed a linear relationship between MHD and incidence of major coronary events, with a 7.4% increase in risk for every 1-Gy increase in MHD [11]. Van den Bogaard [12] used the same prognostic risk factors to build a multivariate Cox regression model: every 1-Gy increase in MHD was associated with a 16.5% increase in the cumulative incidence of acute cardiac events within 9 years after radiotherapy. Those results were consistent with Darby et al.’s observation of a 16.3% increase in risk per 1Gy increase in MHD over the first 4 years of follow-up and a 15.5% increase over 5–9 years after RT. In many studies on radioactive cardiotoxicity, cardiac dose is defined as the average irradiation dose the heart receives [11]. Therefore, we used MHD as a parameter to analyze the dose–response relationship. The new NCCN guidelines require that the MHD be reduced from < 35 Gy to < 26 Gy. In our study, patients with MHD > 20.20 Gy had a higher probability of endpoint events than patients with MHD < 20.20 Gy. Therefore, MHD needs to be further reduced. However, dose distribution to the heart is generally not uniform, and some studies have found that the maximum dose is distributed in the apex or parietal–precordial region and that some parts of the heart are hot spots of >50 Gy [13]. Few studies have focused on anatomical dose distribution in radiation-induced coronary artery disease (RICAD), and it is reasonable to speculate that exposure to RT fields would be associated with an increased risk of RICAD. A Swedish study [14] showed that the stenosis rate of the left anterior descending artery (LAD) was significantly increased in patients who received left-breast cancer RT compared with those who received right-breast cancer RT. This result was related to the distribution of radiation fields and the location of the maximum heart dose in the precordial region, including the LAD. Therefore, MHD is not ideal as a dosimetric parameter reflecting cardiac substructure (especially the LAD) [14].

Walker study [15] is the first study to investigate the associations between breast cancer radiothearpy-induced cardiac doses and subclinical LV dysfunction defined as a GLS reduction > 10%, 6 months after RT. This 6-month follow-up analysis indicated a significant association with mean heart dose, V20 of the heart, mean LV dose and V20 of the LV in univariate analysis. However, they didn’t remain significant in multivariate analysis in particular after adjustment for endocrine therapy.

Firstly, the median follow-up time of our study was 82 days, which is relatively short compared to Walker's 6-month follow-up time. Secondly, our study identified risk factors through the reduction of GLS before, during, and immediately after radiotherapy. During the radiotherapy period, patients did not receive any treatment that damaged or protected the myocardium, so the risk factors we identified are reliable. However, during the follow-up period, we overlooked the issue of patients using aromatase inhibitors, which was a negligence in our experimental design. Walker's study is more comprehensive in this area of work. According to Walker's description in the paper, although the patient did not receive chemotherapy, it was not described whether other treatments such as targeted therapy for Her-2 were performed, as current treatment methods are more diverse. Furthermore, we do not believe that short-term follow-up is meaningless. Firstly, radiation-induced cardiac toxicity is a dose-related toxic reaction, and the probability of toxicity occurring increases with the accumulation of dose. The radiotherapy treatment cycle is relatively long, and during the radiotherapy period, we observed a decrease in GLS, which gave us early indications, and the toxicity may worsen with the radiotherapy process. Of course, long-term follow-up is also important, as survival is an important goal of treatment. We are also observing whether patients who observe a decrease in GLS during radiotherapy will have a worse prognosis compared to those who do not; Secondly, during the 6-month observation period, the heart tissue may undergo self-repair and may undergo treatment activities related to cardiac toxicity, as well as repair, thereby increasing confounding factors.

ΔLVGLS-Epi_post-treatment–pretreatment_ and ΔLVGLS-Epi_post-treatment–pretreatment_/LVGLS-Epi_pretreatment_ were linearly related to V5. LV volume receiving ≥5 Gy (LV-V5) has been shown to be the most important dose–volume prognostic parameter in patients with left-breast cancer and appears to be a better predictor of acute coronary events (ACEs) than MHD [12]. However, we found no linear relationship between V5 and ΔLVGLS_post-treatment–pretreatment_ or ΔLVGLS-Endo_post-treatment–pretreatment_, which might be related to the inclusion of thoracic-tumor patients and only a small number of left-breast cancer patients in our study.

We are also curious about the results that mid- and endo-cardial LV GLS had a different result from epicardial LV GLS in the relationship between the change of LV strains and RT-related parameters. We can’t match a better theory to this phenomenon, whether in anatomical and physiological differences between the endo- and epi- cardial of the heart, or the basic principles of radiotherapy. Perhaps it is a statistical coincidence or bias, but further exploration is necessary.

ΔRVFWLS _post-treatment–pretreatment_ and ΔRVFWLS _post-treatment–pretreatment_/ RVFWLS _pretreatment_ were linearly correlated with MHD and patient age. Age was linearly correlated with decline in systolic function of the right heart compared with the left.

In clinical practice, the systolic and diastolic functions of RV were easily overlooked. Compared with LV, the anatomy and function of the RV are different, and evaluation of its systolic and diastolic functions is still technically difficult. Current guidelines for quantification of RV-function echocardiography recommend the use of multiple indicators to comprehensively describe RV quantitative functions such as TAPSE, RVFAC, RV isovolumic acceleration (IVA), RV Tei index, and s’ [16]. Studies have shown [17]that of patients with left-breast cancer who received RT, 67% had a mean TAPSE reduction of 2.1 ± 3.2 mm (*P* < 0.001), and 39% of patients demonstrated a TAPSE reduction of ≥4 mm. A decrease in s’ was also observed, but it was not statistically significant. In our study, we observed no significant changes in right-heart Tei index or s’, which might be related to our small sample size and the inclusion of patients whose thoracic tumors had other causes.

A recent study showed that in patients with left-breast cancer, the RV free-wall irradiation dose was actually higher than the corresponding LV dose (4.61 vs. 4.37 Gy), despite a lower overall RV than LV irradiation dose (2.85 vs. 4.37 Gy) [18]. Furthermore, in patients with right-breast cancer, the RV was more susceptible to irradiation than the LV (0.45 vs. 0.11 Gy) . In our study, the probability of endpoint events in the right heart was greater than in the left heart, which led to the same conclusion.

Tuonenin's most recent study found that the global longitudinal strain (GLS) was reduced from -18.3 ± 3.1 to -17.2 ± 3.3% (*P* = 0.003) after RT in patients with left-sided breast cancer. Similarly, regional analysis showed a reduction in the apical strain from -18.7 ± 5.3 to -16.7 ± 4.9% (*P* = 0.002) and an increase in basal values from -21.6 ± 5.0 to -23.3 ± 4.9% (*P* = 0.024). Patients with right-sided breast cancer showed deterioration in basal anterior strain segments from -26.3 ± 7.6 to -18.8 ± 8.9% (*P* < 0.001)。Patients with left-sided breast cancer experienced apical impact and global decline, whereas patients with right-sided breast cancer showed basal changes. The regional differences in cardiac impact warrant different methods in screening and in the follow-up of patients with left-sided versus right-sided breast cancer [19]. In our experiment, the probability of right heart dysfunction is greater than that of left heart dysfunction. This may be related to the fact that the patients we included include not only breast cancer patients, but also a large number of lung cancer patients. The dose distribution and radiation field distribution type of lung cancer are different from breast cancer, which may lead to an increase in right heart irradiation, further leading to a decline in right heart function.

RT affects myocardial remodeling, and might be more likely to cause it indirectly. The development of pericardial disease and constrictive pericarditis after RT is particularly important in the remodeling of the RV, which is under relatively low pulmonary-circulation pressure and has thin walls and can therefore be adversely affected by a thickened pericardium [20]. In these cases, pericarditis initially worsens RV diastolic function, increases RV filling pressure, and affects RV systolic function over the course of the disease. This mechanism should not be ignored in the development of RT-mediated RV remodeling [21].

Another important factor is lung injury caused by RT. RT-induced fibrosis does not allow free air exchange in the lungs and causes interstitial pulmonary fibrosis; in addition, RT induces endothelial damage and inflammation in pulmonary microcirculation. Both mechanisms can increase pulmonary resistance and pulmonary-wedge pressure, which in turn can increase pulmonary pressure and ultimately induce or exacerbate RV remodeling [22]. In a large study of 274 adult lymphoma patients [23], RV thickness was minimal (2.7 ± 0.7 mm) in patients treated with anthracyclines and high-dose (>30 Gy) radiation. This suggests that RT induces marked fibrosis and thinning of RV myocardium over a long period of time. In that study’s five groups (control, low-dose anthracycline chemotherapy, high-dose anthracycline chemotherapy, anthracycline chemotherapy + low-dose RT, and anthracycline chemotherapy + high-dose RT), the prevalence of RV systolic dysfunction gradually increased (0.7% vs. 1.5% vs. 4.5% vs. 6.5% vs. 18.4%, respectively; *P* < 0.01) [23].

From a prognostic point of view, assessing RV deformation is very important. Recent studies have shown that RV longitudinal strain (RVLS) is a good predictor of morbidity and mortality in patients with different diseases. A study by Murbraech et al. showed that RVLS and right free-wall strain (RFWS) were significantly reduced in all treated cancer patients [23]. From the control group to patients receiving low- and high-dose anthracyclines to those receiving anthracyclines plus low- and high-dose RT, RVLS gradually decreased (−25.3 ± 2.3% vs. −23.3 ± 3.1% vs. −22.8 ± 3% vs. −21.7 ± 3.1% vs. −21.1% ± 3.6%, respectively; *P* < 0.01). The same was true for RFWS (−30.0 ± 2.6% vs. −27.4 ± 4.3% vs. −27.3% ± 3.9% vs. −26.4 ± 3.9% vs. −25.0 ± 3.8%, respectively).

Although 2D-STE is superior to conventional echocardiography for strain assessment, it has some limitations. The phenomenon of "penetrating planes" makes the 2D-STE technique less valuable than 3D-STE, particularly in anatomically specialized structures such as the right heart [24]. However, it has been reported that there is no difference in longitudinal strain values between 3D and 2D [25, 26]. 2D-STE technology is more widely used than 3D-STE technology in clinical applications for the reason of lower cost and higher patient acceptance. It is easy to promote technology and screen disease. What`s more, 2D-STE is included in our health insurance coverage. Above all, 3D-STE was not used in our study for the examination of right heart function. Of course, we do not exclude more advanced 3D or even 4D technology in future studies.Many diseases such as hypertension, congestive heart failure, and atrial fibrillation are related to aging [27]. These conditions are also known to be associated with fibrosis [28]. Age is thought to play a role in the development of cardiac fibrosis [29]. Compared with left-heart function, pulmonary function is more closely related to right-heart function. After peaking between the ages of 20 and 30 years, lung function gradually declines with age [30]. Furthermore, the effect of lung injury induced by thoracic RT on the RV has been previously elucidated. Based on these facts, it is reasonable to infer that RV function gradually declines with age.

The limitations of our study were as follows:

1. Due to treatment diversification, most patients receive multiple treatments in addition to radiotherapy. We are currently studying only radiotherapy induced myocardial injuries, which makes the enrollment relatively stringent and lead to a small sample size and heterogeneity characterizing the cancer types. Attribute to advances in treatment and increased awareness of health care for cancer patients (e.g., use of cardioprotective drugs), cancer patients are surviving significantly longer nowadays. In addition, multiple factors outside of radiotherapy can affect follow-up outcomes, making long-term follow-up more difficult and a relative short follow-up. In the next stage, we will analyze the tumor-related cardiac injury comprehensively in conjunction with the patients’ subsequent treatments (e.g., chemotherapy or immunotherapy).
2. The heart is a complex geometric structure. We analyzed only the overall long-axis strain of the ventricles using 2D-STE. In the future, radial-strain, circumferential-strain, torsional-function and 3D-STE research can be improved to make results more integral and comprehensive.
3. Our subjects were RT patients with thoracic tumors, and the incidence of radiation pneumonitis was high, resulting in poor image quality.
4. Patients were not classified (such as hypertension, diabetes, or dyslipidemia) for inter group analysis to explore inter group differences.

## Conclusions

1. RT damaged the systolic function of both sides of the heart. Two-dimensional STE could detect damage earlier and more sensitively than conventional echocardiography.
2. The decrease in 2D LV systolic-function parameters between the post- and pre-treatment stages, and the ratio of the decrease to the pre-treatment value correlated linearly with MHD. MHD is an important prognostic parameter for LV systolic function, and V5 may also be an important prognostic parameter.
3. The decrease in 2D RV systolic-function parameters between the post- and pre-treatment stages, and the ratio of the decrease to pre-treatment value correlated linearly with MHD and patient age. MHD and age are important prognostic parameters for RV systolic function.
4. Patients older than 56.5 years were more likely to have right-heart endpoint events than those younger than 56.5 years.
5. Patients with MHD > 20.20 Gy had a greater probability of endpoint events on both sides of the heart than those with MHD < 20.20 Gy.

## Data Availability

The data that support the findings of this study are available from the corresponding author upon reasonable request.

## Abbreviations

2D-STE: Two-dimensional speckle-tracking echocardiography
ACE: Acute coronary event
ANOVA: Analysis of variance
CREC: American cardiac-toxicity Review and Evaluation Committee
DVH: Dose–volume histogram
FAC: Fractional-area change
FWLS: Free-wall longitudinal strain
IVA: Isovolumic acceleration
LAD: Left anterior descending artery
LV: Left ventricular
LVEF: Left ventricular ejection fraction
LVGLS: Left ventricle global longitudinal strain
LVGLS-Endo: LVGLS of the endocardium
LVGLS-Epi: LVGLS of the epicardium
MAPSE: Mitral annular-plane systolic excursion
MHD: Mean heart dose
NCCN: National comprehensive cancer network
PSV: Peak systolic velocity
RFWS: Right free-wall strain
RICAD: Radiation-induced coronary artery disease
RIHD: Radiation induced heart disease
ROCs: Receiver operating curves
RT: Radiotherapy
RV: Right ventricular
RVLS: Right ventricular longitudinal strain
s’: Peak systolic velocity of the free wall of the tricuspid annulus
SD: Standard deviation
STE: Speckle-tracking echocardiography
TAPSE: Tricuspid annular plane systolic excursion
Tei: Cardiac Tei index
V5: Percentage of heart volume exposed to 5 Gy or more
V10: Percentage of heart volume exposed to 10 Gy or more
V20: Percentage of heart volume exposed to 20 Gy or more
V30: Percentage of heart volume exposed to 30 Gy or more
V40: Percentage of heart volume exposed to 40 Gy or more
YI: Youden index
Δ: The difference between
